# Severe Fever with Thrombocytopenia Syndrome, South Korea, 2012

**DOI:** 10.3201/eid1911.130792

**Published:** 2013-11

**Authors:** Kye-Hyung Kim, Jongyoun Yi, Gayeon Kim, Su Jin Choi, Kang Il Jun, Nak-Hyun Kim, Pyoeng Gyun Choe, Nam-Joong Kim, Jong-Koo Lee, Myoung-don Oh

**Affiliations:** Seoul National University College of Medicine, Seoul, South Korea (K.-H. Kim, G. Kim, S.J. Choi, K.I. Jun, N.-H. Kim, P.G. Choe, N.-J. Kim, J.-K. Lee, M.-D. Oh);; Pusan National University School of Medicine, Busan, South Korea (J. Yi)

**Keywords:** severe fever with thrombocytopenia syndrome, phlebovirus, South Korea, viruses, bunyavirus

## Abstract

We report a retrospectively identified fatal case of severe fever with thrombocytopenia syndrome (SFTS) in South Korea from 2012. SFTS virus was isolated from the stored blood of the patient. Phylogenetic analysis revealed this isolate was closely related to SFTS virus strains from China and Japan.

Severe fever with thrombocytopenia syndrome (SFTS) causes signs and symptoms including high fever, vomiting, diarrhea, thrombocytopenia, leukopenia, and multiple organ failure and has a 6%–30% case-fatality rate (*1*–*4*). Caused by a novel bunyavirus, SFTS virus (SFTSV), SFTS was initially reported in China in 2011 (*1*). SFTSV has been detected in *Haemaphysalis longicornis* ticks, which have been implicated as a vector of the virus (*1*). *H. longicornis* ticks widely inhabit the Korean Peninsula (*5*,*6*), and the Korea Centers for Disease Control and Prevention reported that SFTSV was detected in samples from *H. longicornis* ticks collected during 2011–2012 in South Korea (*7*). Seroconversion and viremia of SFTSV have been demonstrated in domesticated animals such as goats, sheep, cattle, pigs, and dogs; these animals have been implicated as intermediate hosts in SFTSV-endemic areas (*8*,*9*). SFTSV was also detected in Japan in February 2013 (*10*). We report a retrospectively identified case of SFTS in South Korea from 2012 and the characterization of the SFTSV isolated from the patient.

## The Study

On August 3, 2012, fever developed in a previously healthy 63-year-old woman who lived in Chuncheon-si, Gangwon Province, South Korea; the same day, she noticed a lump on the left side of her neck. She visited a local clinic, and ciprofloxacin and ceftriaxone were started on the first day of illness. The patient reported that, 2 weeks before her fever started, she noticed an insect bite on her neck while she was working on a crop farm in Hwacheon-gun, Gangwon Province (in the northernmost part of South Korea). She did not recall having contact with any domestic animals on the farm and had no history of travel outside South Korea in the month before illness onset. 

On the third day of her illness, she began having watery diarrhea, 6 times per day. On the fourth day of the illness, thrombocytopenia and leukopenia were recorded at the local clinic (Table). Because of worsening thrombocytopenia, she was transferred to another hospital. Ciprofloxacin was changed to doxycycline, and ceftriaxone was continued. A computed tomography scan of the neck showed an enlarged (1.6 cm), necrotic lymph node. Multiple lymph nodes on the left cervical and left axillary areas were also swollen. On the sixth day, the patient was transferred to Seoul National University Hospital.

**Table:** Laboratory findings of the patient with severe fever with thrombocytopenia syndrome, South Korea, 2012*

Laboratory test (reference range)	Day 2	Day 4	Day 5	Day 6	Day 7	Day 8	Day 9	Day 10
Hematocrit, % (36–48)	NA	NA	30	30	38	32	34	20
Hemoglobin, g/dL (12–16)	12.9	13.6	12.4	12.4	12.9	12.8	9.9	7.0
Leukocytes, cells/μL (4,500–10,000)	1,800	1,300	1,600	1,600	2,100	3,150	4,300	4,700
Neutrophils, % (50–75)	47	60	56	51	76	35	32	33
Lymphocytes, % (20–44)	36	36	39	41	18	58	57	62
Atypical lymphocytes, % (0)	NA	NA	NA	NA	4	5	8	3
Platelets, /μL (130,000–400,000)	136,000	98,000	50,000	25,000	32,000	70,000	159,000	116,000
AST, IU/L (0–40)	56	NA	180	383	537	1059	2279	NA
ALT, IU/L (0–40)	32	NA	66	115	137	199	403	NA
Creatine kinase, IU/L (20–270)	NA	NA	NA	5,127	6,966	7,830	15,224	NA
LDH, IU/L (100–225)	NA	NA	NA	NA	NA	5270	NA	NA
Creatinine, mg/dL (0.7–1.4)	NA	0.60	0.70	NA	0.59	0.99	2.17	3.01
aPTT, sec (26–35.3)	NA	NA	NA	44.1	45.4	75.6	71.6	>400
Prothrombin time, INR (0.8–1.2)	NA	NA	NA	0.98	0.99	1.08	1.06	>25
Fibrinogen, mg/dL (230–380)	NA	NA	NA	172	151	136	124	57

*Day 1 was the onset day of the illness. Platelets were transfused on day 7 and day 9, and fresh frozen plasma was transfused on day 9 and day 10. Creatinine kinase-MB levels were 7, 5, 25.7, 117.5 and 186.2 ng/mL on days 6, 7, 8, 9, and 10, respectively (reference range 0.6–6.3 ng/mL). No anticoagulants were used during the hospitalization and for the renal replacement therapy. NA, not available; AST, aspartate aminotransferase; ALT, alanine aminotransferase; LDH, lactate dehydrogenase; aPTT, activated partial prothromboplastin time; INR, international normalized ratio.

At admission to the hospital, the patient was febrile but alert. Her temperature was 38.7°C, blood pressure 126/70 mm Hg, heart rate 86 beats per minute, and oxygen saturation 92% on room air. Her face was puffy, with a sunburned appearance, and both conjunctivae were congested. The insect bite site on her posterior neck was swollen and erythematous, and the draining cervical lymph node was enlarged. Petechiae were observed on her shoulders and lower extremities.

Laboratory test results showed pancytopenia and elevated serum aminotransferase levels; prothrombin and activated partial thromboplastin times were normal, but fibrinogen level was decreased (Table). A urine dipstick test showed albuminuria (+++), and microscopic examination of the urine revealed >100 erythrocytes per high-power field. Test results for antibodies against *Orientia tsutsugamushi*, Hantaan virus, and leptospira were negative. A chest radiograph showed bilateral increased vascular markings, and the plasma level of B-type natriuretic peptide increased to 134 pg/mL (reference range <100 pg/mL); these findings suggested cardiac dysfunction. 

On the eighth day of her illness, the patient spoke incoherently and was unable to communicate. Cerebrospinal fluid analysis showed no erythrocytes or leukocytes and a normal chemistry profile. A computed tomography scan of the brain showed no evidence of hemorrhage or infarction and no other abnormalities. She was transferred to the intensive care unit. On the ninth day, she was intubated and placed on continuous renal replacement therapy. On the tenth day of illness (August 12, 2012), the patient died of multiple organ failure. Ceftriaxone and doxycycline were continued until the patient’s death. Antiviral drugs, corticosteroids, immunosuppressive agents, or intravenous immunoglobulin were not given.

**Figure 1:**
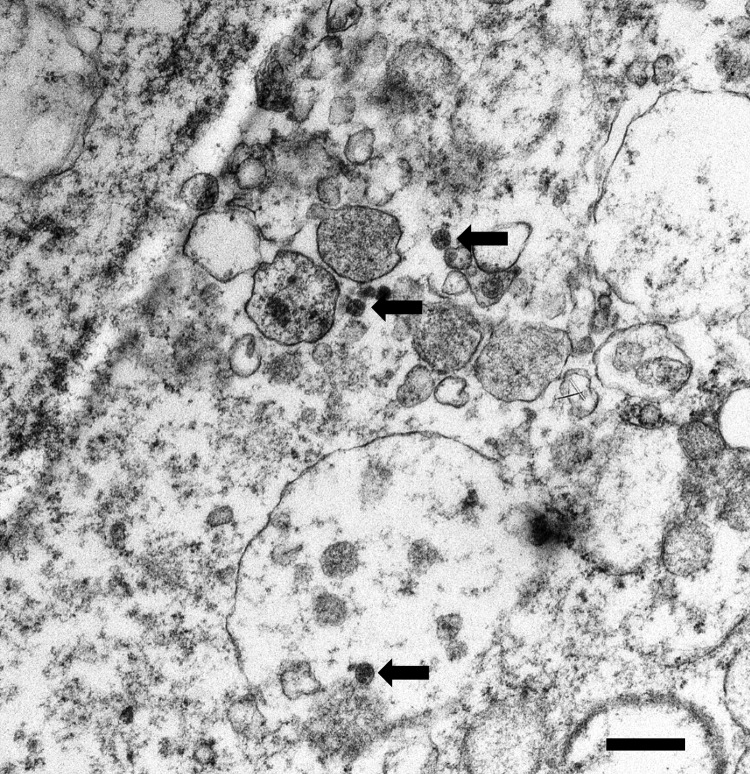
Transmission electron microscopy image of Vero cells infected with severe fever with thrombocytopenia syndrome virus (arrows). Scale bar indicates 500 nm.

Because viral infection was suspected but no virus could be identified, an anticoagulated blood sample was obtained from the patient on the eighth day of illness and stored at −70°C. When testing for SFTSV became available 7 months later, we inoculated monolayers of Vero cells with the patient’s blood sample and cultured the cells at 37°C in a 5% carbon dioxide atmosphere. A culture supernatant obtained 13 days after the inoculation was used for genetic analysis. The culture supernatant was also used to inoculate DH82 cells when the cell line became available; 5 days after the inoculation, we observed a cytopathic effect of SFTSV in DH82 cells. The SFTSV-infected Vero cell monolayer was fixed according to described methods (*11*) and cut on ultramicrotome (RMC MT-XL) at 65 nm. Ultrathin sections were stained with saturated 4% uranyl acetate and 4% lead citrate before examination with a transmission electron microscope (HITACHI-7100; Hitachi High-Technologies, Ibaraki, Japan) at 75 kV (Figure 1).

RNA was extracted from the stored blood and from virus-infected Vero cells by using a QIAamp Viral RNA Mini Kit (QIAGEN, Hilden, Germany). Reverse transcription PCR (RT-PCR) was performed to amplify the partial large (L) segment of the viral RNA from the stored blood to confirm SFTSV, as described (*12*). RT-PCR results were positive, and direct sequencing was done. A BLAST search (http://blast.ncbi.nlm.nih.gov/Blast.cgi) showed no sequences from organisms other than SFTSV.

**Figure 2:**
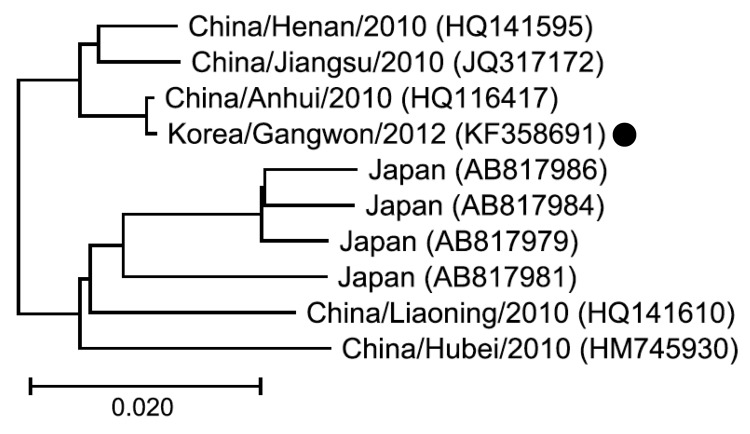
Phylogenetic tree for the RNA-dependent RNA polymerase (RdRP) gene sequences of the large segment of an isolate obtained from a patient in South Korea who died of an illness retrospectively identified as severe fever with thrombocytopenia syndrome (SFTS) (black dot) compared with representative SFTS virus strains from China and Japan. The tree was constructed on the basis of the nucleic acid sequences of the RdRP genes by using the neighbor-joining method. Location, year of isolation, and GenBank accession numbers are indicated. Branch length of the tree shows the evolutionary distance. Scale bar indicates 2.0% sequence distance.

Using the culture supernatant, full lengths of all 3 genome segments (L, medium [M], and small [S]) were sequenced by RT-PCR and direct sequencing was performed by using primers designed from previously published SFTSV sequences. After polyadenylation of 3′ ends of the genomic and complementary RNAs, the sequences of the segment ends were obtained by rapid amplification of cDNA ends. The complete sequences of the L, M, and S segments were deposited in GenBank (accession nos. KF358691–KF358693). Sequences that had homology to our isolate were identified by BLAST search. The L, M, and S segments of the isolate showed 95.8%–99.8%, 94.1%–99.9%, and 94.8%–99.7% identity, respectively, to previously reported SFTSV sequences. We also constructed a phylogenetic tree by the neighbor-joining method using RNA-dependent RNA polymerase gene nucleic acid sequences to compare the isolate we obtained to representative SFTSV strains from China and Japan; the isolate and the other strains were closely related (95.9%–99.9% sequence relatedness) but not identical (Figure 2).

## Conclusions

We confirmed a case of SFTS in South Korea in 2012 by isolation of SFTSV from a stored blood sample collected shortly before the patient’s death. The patient had a history of an insect bite while working on a crop farm in Hwacheon-gun, Gangwon Province, the northernmost part of South Korea. Phylogenetic analysis of the RNA-dependent RNA polymerase gene showed that our virus isolate was closely related to SFTSV strains reported from China and Japan. 

As of July 5, 2013, the Korea Centers for Disease Control and Prevention had confirmed 13 cases of SFTS by RT-PCR; of these patients, 8 were dead and 5 alive (*13*). Except for our patient, who died in 2012, all cases occurred during 2013.
